# Historical perspective of neurology in Iran

**Published:** 2019-01-05

**Authors:** Shri Kant Mishra, Hadi Mohammad Khanli, Golnoush Akhlaghipour, Ghazaleh Ahmadi Jazi, Shaweta Khosa

**Affiliations:** 1 Department of Neurology, David Geffen School of Medicine, University of California Los Angeles, Los Angeles, United States; 2 Department of Neurology, George Washington School of Health and Medical Sciences, Washington, DC, United States

**Keywords:** Iran, Neurology, Medicine, History

## Abstract

Iran is an ancient country, known as the cradle of civilization. The history of medicine in Iran goes back to the existence of a human in this country, divided into three periods: pre-Islamic, medieval, and modern period. There are records of different neurologic terms from the early period, while Zoroastrian (religious) prescription was mainly used until the foundation of the first medical center (Gondishapur). In the medieval period, with the conquest of Islam, prominent scientists were taught in Baghdad, like Avicenna, who referred to different neurologic diseases including stroke, paralysis, tremor, and meningitis. Several outstanding scientists developed the medical science of neurology in Iran, the work of whom has been used by other countries in the past and present. In the modern era, the Iranian Neurological Association was established with the efforts of Professor Jalal Barimani in 1991.

## Introduction

The Middle East, especially Iran, is known as the cradle of civilization. Iran is an ancient Middle Eastern country, known as Persia until 1935 with a vast land including all of today’s Iraq, Lebanon, Jordan, Armenia, Turkey, and Syria, as well as parts of Saudi Arabia, Afghanistan, Pakistan, the Caucasus, Central Asia, and Egypt.^   1 ^^,^^   2 ^ What is known today as Iran is a country with about 1.7 million km^2^, located in the south of the Caspian Sea and north of the Persian Gulf and the Gulf of Oman, bordering with Iraq, Turkey, Azerbaijan, Turkmenistan, Armenia, Afghanistan, and Pakistan.^   3 ^

The Persian Empire was formed in the 6^th^ century BC by migration of two Aryans, including the Medes and the Persians. The different empires Persia experienced including Achaemenid and Sasanian empires, Safavid, Afsharid, and Qajar dynasties and the invasions to and from the neighboring countries made not only variations in the country’s land, but also in the culture.^   4^

Throughout history, Persia was one of the prominent academic centers in various scientific fields including medicine, astronomy, mathematics, and philosophy.^   5 ^ The Persians have always been great contributors to the field of medicine, and the Persia empire was known for having the most renowned physicians such as Abu Bakr Muhammad ibn Zakariya Al-Razi (Rhazes, 865–925 AD), Ali ibn Al-Abbas-al-Majusi (Haly Abbas, 949–982 AD), Abu Ali Al-Hussain ibn Abdullah ibn-e-Sina (Avicenna, 980–1037 AD), and Zinn-ol-Abedin Esmail Jorjani (Sorsanus, 1042–1137 AD).^   6 ^^-^^   9 ^ Some of their contributions in medicine including the texts of Razi’s Kitab al-Hawi (The Continent) and Qanoon-fel-teb (The Canon) by ibn-e-Sina were the core of western medicine from the 13^th^ to the 19^th^ centuries.^   10 ^^-^^   13^

Persians also established successful academic centers such as the Academy of Gondishapur (3^rd^ century AD), which was the first teaching hospital in which medical students were trained under the supervision of physicians.^   14 ^^,^^   15 ^ According to some sources, Gondishapur University was the first whole hospital system in the world.^8^

The idea of xenotransplantation was proposed during the days of Achaemenids (550–330 BC) and its evidence is still present in Persepolis.^   16 ^ Knowledge of anesthesia was illustrated in Shahnameh, written by the Iranian poet of 10^th^ century, Ferdowsi, who described using a particular wine as a sedating agent for performing a cesarean section.^   17 ^^,^^   18 ^ Later in the 10^th^ century, the first comprehensive medical textbook in Farsi language, Hidayat al-Muta’allemin Fi al-Tibb, was written by Abu Bakr Joveini, who was one of the students of Abu Bakr Muhammad ibn Zakariya Al-Razi.^   19 ^^,^^   20 ^ Modern academic medicine was established in Iran in 1878 when Dr. Joseph Plumb Cochran founded the first contemporary hospital (Westminster Hospital) and medical college in Urmia.^   21 ^

Over the Persia’s turbulent centuries, numerous texts and valuable books of Iranian scientists were demolished following the invasion of the Macedonians (Alexander), Arabs, Tatars, and Afghans; thus, there are not many available records regarding the development of medicine in ancient Iran.^   22 ^ The present review aims to outline the most prominent milestones in the development of neuroscience and neurology in Iran, as well as mentioning the most significant contribution of Iranian scientists to the world of medical science.

**A brief history of medicine in Iran**

The history of medicine in Iran goes back to the existence of a human in this country. Although medicine existed since the humans existed in one land, it is difficult to track the actual origin of medicine in the early period. What is known is that medicine was always mixed with religion in this country.^   23 ^ History of medicine in Iran can be divided into three periods: pre-Islamic (early), medieval (Islamic), and modern period. 

Among the primary tribes migrating to the Persia, the Magis, one of the tribes of Medes, were expert in astrology, herbal medications, and magical acts, who later formed the Zoroastrian hereditary priesthood, known as the mobeds (the healers of the soul) and directed the ritual religious remedies.^   24 ^ The books of Avesta (the main collection of Zoroastrianism holy texts), which were written during the 6^th^ century BC and attributed to Zoroaster himself, are considered as the earliest texts in Farsi that address health and diseases.^   22 ^ The Vendidad (Videvdad), which literally means “The law against demons”, is the most popular surviving text of Avesta, which was probably written at the beginning of the Arsacid period (around 200-250 BC), and contains some records of the ancient Persian medicine.^   25 ^ In the Vendidad, three kinds of medicine were described: medicine through the knife (surgery), through the plants (herbal medicine), and through the divine words (cure by prayers); the latter was the most important one as they believed that sickness was the consequence of demonic forces.^   23 ^ The close observance of medical ethics and rules can be seen in the records from Avesta, like punishments for surgical errors.^   26 ^ In addition, several operations like cesarean section are reported from that period.^   27 ^ Anatomy, pathology, and physiology were practiced using animal models in Babylonian schools.^   28^

**Figure 1:**
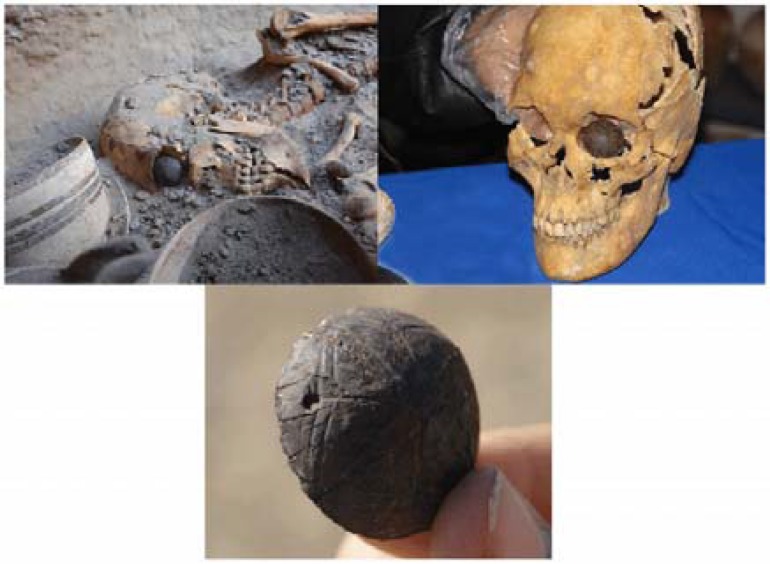
The earliest artificial eye found by Iranian archaeologists in Shahr-e-Sukhteh, Iran

Burnt City (Shahr-e Sukhteh) which is located in Sistan va Baluchestan Province, southeast of Iran, was recently recognized as mainland, Iran’s largest prehistoric site.^29^ A unique discovery was the result of excavations in 2006 when archaeologists found an artificial eyeball on a female skeleton in an ancient grave of the Burnt City’s cemetery, dated back to 4800 years ago.^29^^,^^   30 ^ The face, reconstructed by a group of Iranian and Italian researchers and displayed in Rome’s National Museum of Oriental Art in 2010, carries the first ocular prosthesis ever made by man which has been worn during its owner’s lifetime^29^ (Figure 1).^   31 ^

In addition, the first ancient cranial surgery was conducted in the 3rd century BC, in the Burnt City, where archaeologists found a skull of a 13-year-old girl with congenital hydrocephalus, who underwent surgery for removing a part of her skull.^   32 ^

Based on the investigations, the patient lived for more than six months after the surgery.^   32 ^

**Figure 2:**
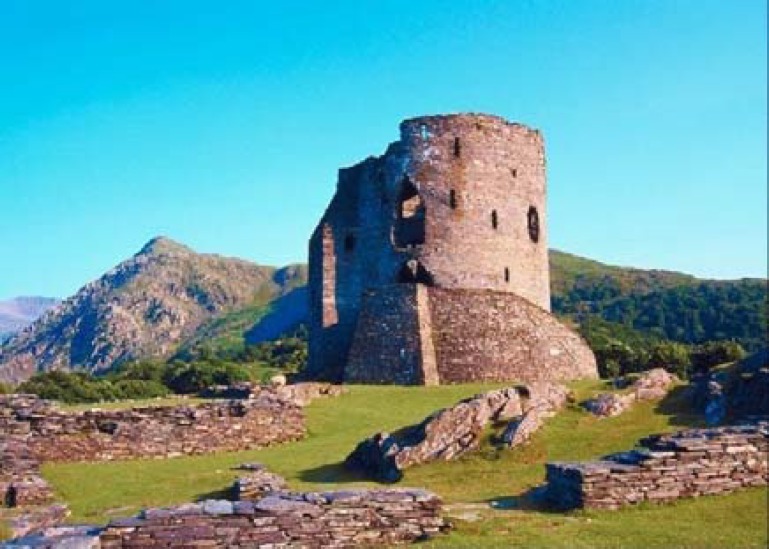
Ancient Gondishapur medical university, the most important medical center of the ancient world during the 6^th^ and 7^th^ centuries

By the foundation of the academic medical center, Gondishapur (271 AD) (Figure 2)^   33 ^ during the Sasanian period, different scholars were trained. By its peak of success during Anoushirvan’s time (Khosrow II), the physicians gathering in the form of a symposium and translating Greek, Indian, and Chinese books in its library developed different aspects of medicine.^   34 ^ Although the quotes of kings at that time indicate the significant role of Gondishapur in development of science, little is known about the details, perhaps due to the destruction of libraries by the invasion of Arabs to this country.^   34 ^

By the introduction of Islam to Iran (651 AD), in the medieval period, there were significant improvements in hygiene observation methods.^   35 ^ The Gondishapur medical center continued its activity until a few centuries later, training the first Muslim physician, Al-Harith ibn Kalada. However, after the conquest of Islam, the Zoroastrian healing methods (divine words) diminished and the destructed libraries of Gondishapur medical center was replaced with the Holy Koran.^   35 ^ After a few years, the medical center of the country was transferred to Baghdad, where the Greek, Iranian, and Indian scientists gathered to develop medicine based on the vast scientific works of outstanding Persian scientists (including Razi or Rhazes, Alpharabius, Jorjani, and Avicenna) to introduce their medical knowledge to the Islamic world.^   35 ^

**Figure 3:**
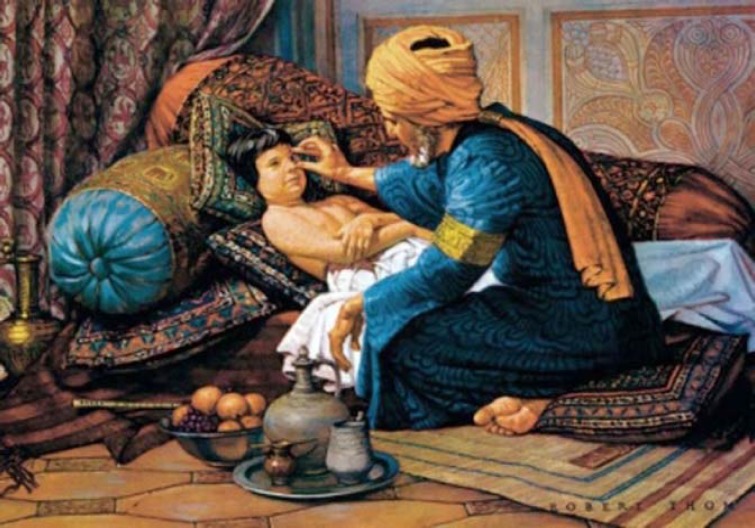
Rhazes (865-925 AD)

**Figure 4:**
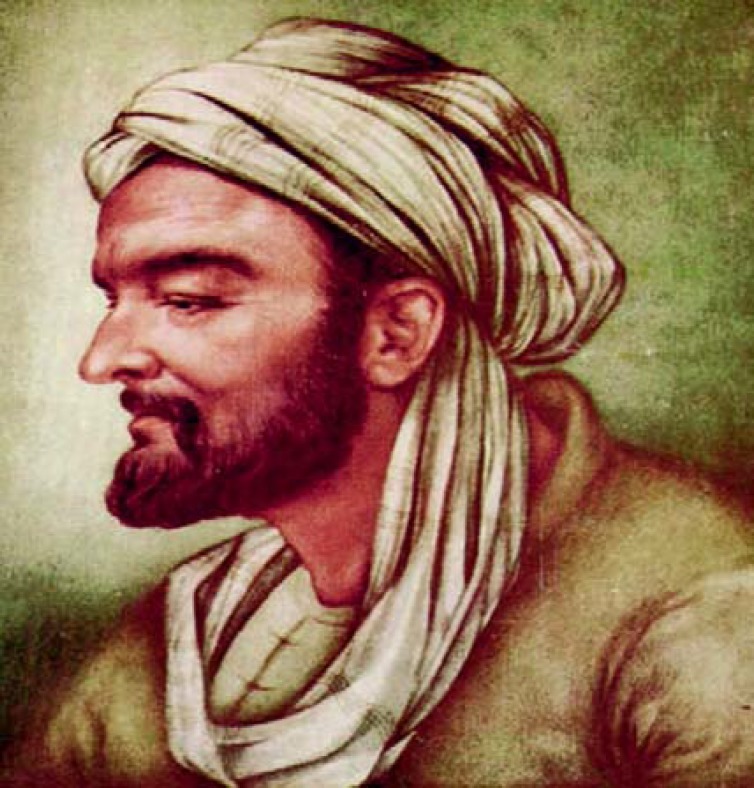
Ibn Sina (Avicenna) (980-1037 AD)

Avicenna used physical examination and history taking in his diagnostic procedure and his great medical book, named “The Canon of Medicine”, was the main reference of medicine in the western countries until the 16^th^ century.^   9 ^ Zakariya al-Razi was an alchemist before his medical studies who took a significant step in the Persian pharmacology.^   36 ^ Rhazes, the great chemist, mathematician, physician, and astronomer was the first to challenge previous scientists like Galen and reject their errors, like the existence of a bone in heart base.^   28 ^ Rhazes (Figure 3)^     37 ^ and Avicenna (Figure 4)^   38 ^ as well as Al-Majusi Ahvazi also reported cancer, the different types and anti-cancer herbal medicines, and surgical treatment for its removal.^39^ The most significant advances in medicine have been recorded at this period by the great work of these scientists; Iranian reports indicate the diagnosis of several diseases at that time like Leishmaniasis,^   40 ^ facial palsy and spasm,^   41 ^ and anatomy.^   42 ^

**Figure 5:**
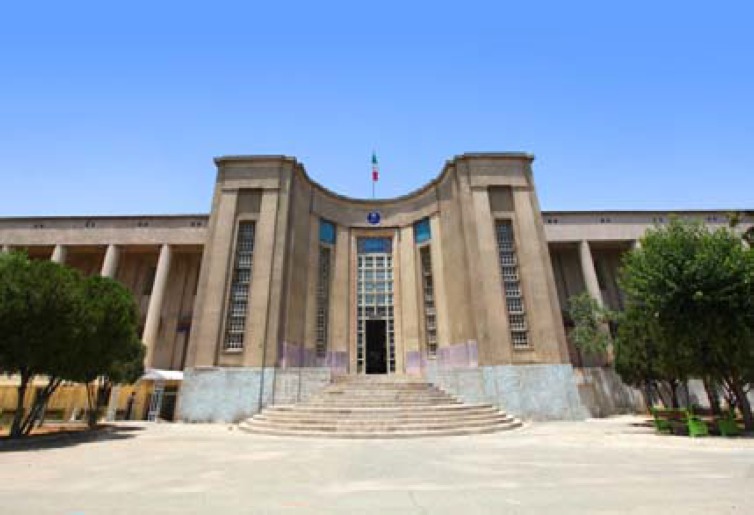
Tehran University of Medical Sciences

Finally, modern medicine started with the establishment of Dar al-Funun in 1851 for training physicians academically under the supervision of European and American teachers.^   43 ^ This was the first time the fields of medicine was separated, and the primary rules of each specialty were mainly derived from the European medical schools; some scholars completed their medical education in European countries.^   43 ^ Given said that, all the achievements of Iranian physicians and scientists were separated as traditional medicine and considered as a subsidiary field. University of Tehran, Tehran, Iran, was the first modern university in Iran which was established in 1934 and considered as the mother university in Iran (Figure 5).^   44 ^ The School of Medicine of Tehran University was established in 1934 which is still the most well-known center of medical education in Iran.^   44 ^

**History of neurology in Iran: Neurology in the pre-Islamic period**

As described above, the history of medicine in Iran is divided into three periods. In the earliest evidence about medical practices, which was found from the library of Ashurbanipal at Nineveh (668 BC), Assyria, and from cuneiform tablets from the city of Uruk (2500 BC), Sumer, two texts regarding the “prescriptions for disease of the head” show the origin of neurology in the Persia, which included descriptions of signs and symptoms of important neurologic disorders such as different types of pain, seizure, movement disorder, stroke, cranial nerves involvement, Parinaud’s syndrome, head trauma and spinal injuries, headaches, spinal muscular atrophy, dementia, and coma.^   45 ^

The most prominent progression of science and medicine happened during the period of the Sasanian dynasty in major learning centers such as Gondishapur, which reached its peak during Anoushirvan’ time.^   34 ^ Although the foundation of Gondishapur and its importance in training significant scholars in medicine are mentioned in history, there is little information about the details of the changes in medicine during this period. 

**Neurology in the medieval period**

In the medieval Iranian medical texts, “The Canon of Avicenna” and “Al-Hawi of Rhazes”, the terms “Sare” (falling sickness), “Ommo-ol-sabyan” (children epilepsy), and “maraze-el-kaheni” (diviners’ illness) have been used for epileptic attacks. Medieval Iranian medical doctors recommended multi-disciplinary approaches for the treatment of epilepsy such as diet therapy, electrical-shock therapy, phlebotomy, and antiepileptic medicaments like Lavandula stoechas, Pimpinellaansium, and Anacyclus pyrethrum, essential oil of Valeriana officinalis.^   9 ^^,^^   36 ^ The anticonvulsive effects of these medications have been verified by recent animal studies.^   46 ^

“Seda” (simple and non-recurrent headaches), “bayzeh” (a recurrent and bilateral headache), and “shaqhiqheh” (a recurrent and unilateral headache”, migraine) were terms used by medieval Iranian practitioners for classification of headaches, found in “The Canon of Avicenna”.^   46 ^ Avicenna believed migraine origins to be skull bone, intra-parenchymal, skull underneath membrane (dura mater), extracranial, or brain and meninges (pia-mater).^   47 ^ The prescribed drugs of Avicenna to cure a severe headache included anti-neuroinflammatory agents and topical narcotics like opium poppy.^   47 ^ The main route for administration of drugs to affect the central nervous system (CNS) was a nasal route.^   48 ^

The term “Sekteh” in “The Canon of Avicenna” has been used to describe a stroke; signs and symptoms of stroke such as hemiplegia, asphyxia, dizziness, vertigo, impaired vision, tremor, and anxiety have been described in detail by Avicenna.^   46 ^ Medieval Iranian physicians used medicinal plants and herbs such as beetroot, citrus fruit, and ginger in the management of ischemic stroke, the effects of which have been proven by recent clinical trials.^49^ Avicenna defined the Willis’ circle of brain six centuries before it was described in modern medicine for the first time.^   50 ^

Tabari first described paralysis and tremor in his work named “Ferdos-ol Hokame”.^   51 ^ After a few years, Rhazes separated seven cranial nerves and 31 spinal nerves for the first time; he used neuroanatomy in the localization of nervous system lesions and correlated them with clinical signs.^   51 ^ In his sixth book of “Al-Hawi”, Rhazes completely described the anatomy of the facial and cranial nerves as well as the spastic and paralytic disorders, later completed by Avicenna and Jorjani.^   41 ^ Also, hydrocephalus and spinal disorders were described by Avicenna in “The Canon of Avicenna”.^   52 ^^,^^   53 ^ Avicenna’s treatment for spinal trauma included food and drug therapy, massage, phlebotomy, cupping, dry sauna, and surgery.^   54 ^

**Neurology in the modern period**

Before the end of the Qajar dynasty, the physicians were trained by their masters and visited patients at their office or the patients’ bed.^   12 ^ The great scientist-physicians of the medieval period, like Avicenna and Rhazes, were expert in different aspects of science including medicine.^   12 ^ Very few physicians were trained in western countries, and the primary medical knowledge of physicians originated from their examinations and discoveries and seminars with other scientists in the country.^   12 ^ The first western-educated Iranian physician, Mirza-Baba, graduated in modern medicine from the United Kingdom (UK) in 1812, which was the door to the education of students abroad.^   55 ^

After the establishment of Dar al-Funun by efforts of Mirza Taghi-Khan Farahani, known as Amir-Kabir, the Prime Minister of Naser al-Din Shah in 1851, the first modern training of medicine started in 1918 by European teachers and trainers.^   43 ^ In the next few years, about 50 students were sent to France for studying medicine, which included the first graduates of Tehran Dar al-Funun School; in 7 years (1928-1935), about 640 students, including 125 medical students, were sent to European countries by the government’s expenses.^   56 ^ This trend continued after the establishment of University of Tehran in 1934, which changed the face of medical sciences in Iran, using the western medical science as the reference.^   57 ^ The physicians returned to their homeland and took the main scientific roles, including the establishment of Farhangestan-e-Awwal (First Academy) in 1935, responsible for the translation of international medical textbooks to Persian.^   57 ^ The activity of the Second Academy continued until 1979 (the cultural revolution in Iran and the change of government to the Islamic Republic).^   57 ^ The Third Academy consisted of four separate organizations for Farhangestan-e Olum (Sciences, 1987), Farhangestan-e Olum-e Pezeshk (Medicine, 1989), Farhangestan-e Zaban va Adab-e Farsi (Persian Language and Literature, 1991), and Farhangestan-e Honar (Art, 1998).^   57 ^ A significant change after the revolution in medicine could be the fact that physicians who traveled abroad to learn medicine were not sponsored by the government and most of them did not come back to Iran to share their knowledge and experience with Iranian students.^   57^

**Figure 6:**
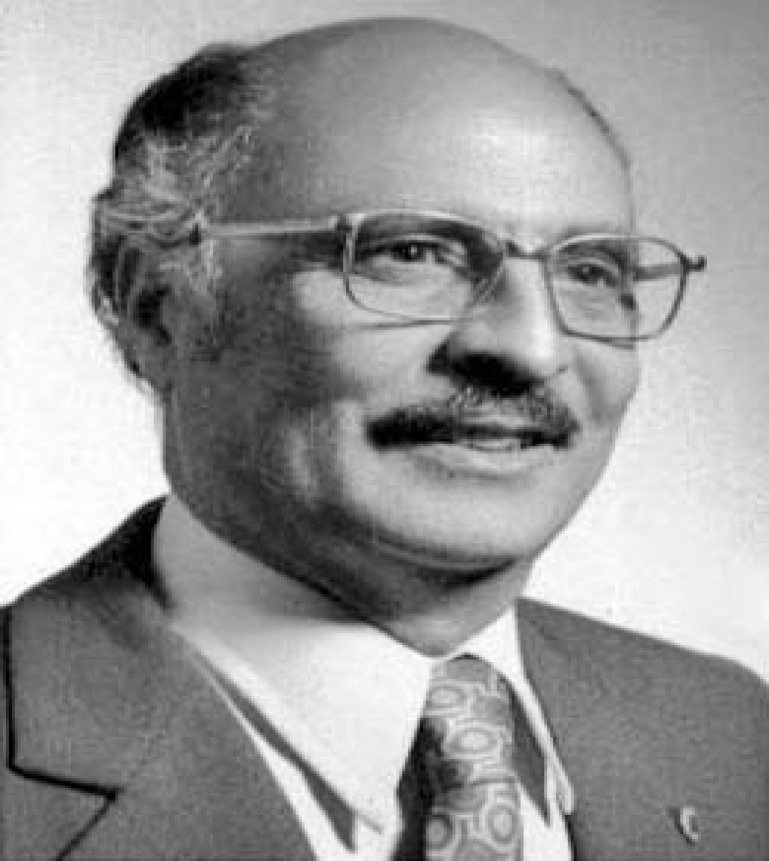
Professor Seyed Ebrahim Chehrazi, the father of modern neuropsychiatry in Iran

In 1929, Dr. Ebrahim Chehrazi (Figure 6),^         58 ^ “The father of modern neuropsychiatry in Iran”, was chosen by the government for a scholarship to go to Paris to study medicine. 

After finishing medical school with specialized training in neurology and medical pathology, he returned to Iran and started teaching at the newly-established medical school in Tehran where he created and chaired the Department of Neurology at Tehran University of Medical Sciences with three clinical divisions (out-patient clinics, neurophysiology, and neurosonology) and 130 beds.^59^ Ebrahim Chahrazi, between 1936 and 1968, wrote two books in French and more than ten books on psychiatry, disease, and health, and published thirty-six articles in the Medical School Magazine and eight articles in other journals.^     60 ^

**Figure 7:**
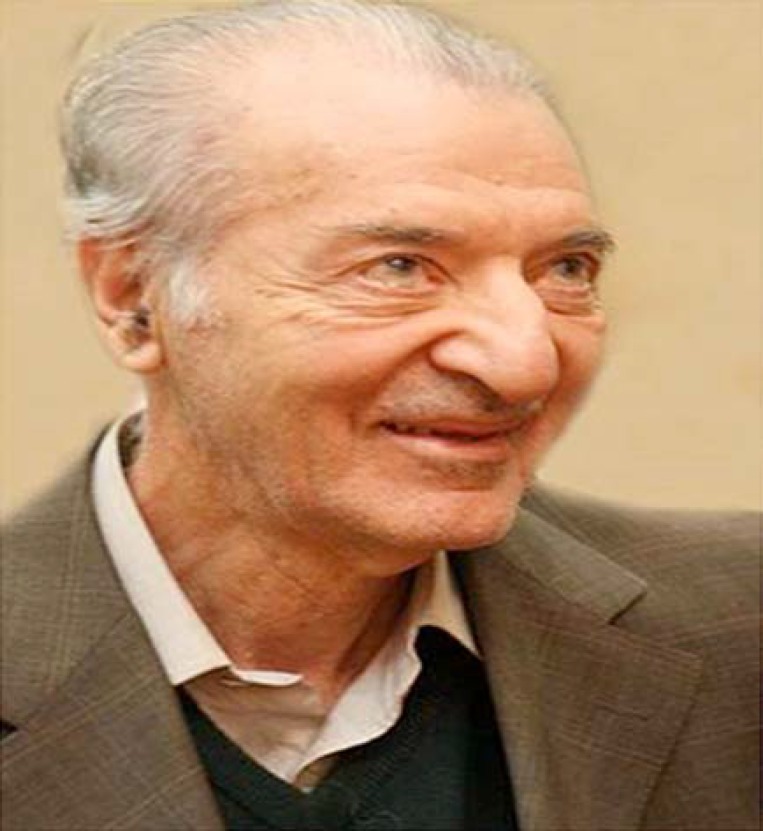
Professor Jalal Barimani (1929-2012), the founder of Iranian Neurological Association

The units of Hospital of Tehran University of Medical Sciences were separated as cardiology, nephrology, rheumatology, and neurology.^   61 ^ In 1991, Professor Jalal Barimani (Figure 7)^     62 ^ established the Iranian Society of Neurology, later named as Neurological Association (in 2003), as the leading scientific neurological society in Iran, which hosts the annual Iranian neurological congress.^   63 ^ Dr. Barimani authored more than 200 scientific papers and books in fields of neurology, neurophysiology, and literature in Farsi, English, and French.^     62 ^ The Iranian Journal of Neurology, the first neurologic journal in Iran, is dedicated to the same association.^   64 ^

In 2003, the Iranian Center of Neurological Research (ICNR), located in Imam Khomeini Hospital in Tehran City, was established as the first neurological research center in Iran by Professor Majid Ghaffarpour and in 2004, Neuroscience Research Center (NRC), located in Shahid Beheshti University of Medical Science campus in Tehran, was established under the leadership of Dr. Fereshteh Motamedi.

Currently, there are 12 centers with neurological residency programs in Iran (in Imam Khomeini, Shariati, and Sina hospitals) in 4-year course, training about 93 residents annually, while the number of neurology fellowship programs is limited. Recently, Tehran University of Medical Sciences has established fellowship programs in sleep disorders and multiple sclerosis (MS). 

Although many hospitals in Iran, especially in the capital (Tehran), have a neurology unit, the number of neurology hospitals is few. Professor Majid Samii, distinguished Iranian neurosurgeon and medical scientist,^      65 ^ has established the 400-bed neurosurgery hospital in Rasht, North of Iran, in 2012, and is seeking to establish another in west of Tehran. Nonetheless, there is still a need for neurology hospitals and research centers in this country. Approximately 950 neurologists are currently working in Iran under the supervision of the health care system (one neurologist per 900000 Iranian citizens).^   66 ^ Although several steps have been taken to increase the number of neurosurgeons, one of the obstacles raised is the number of years for neurosurgery residency, which calls for greater attention to this medical specialty.^   67 ^

Even though neuroscience is a relatively new specialty in this country, Iranians have taken great contributions in international neuroscience, publishing several papers, mainly in neuropharmacology subfield, and fewer papers in neurohistory, psychopharmacology, and artificial intelligence.^   68 ^ Another achievement of Iran is the development of neuroimaging methods in the country; as reported, more than 88% of magnetic resonance imaging (MRI) is performed by neurosurgeons, orthopedists, and neurologists in the country and the most common organs scanned include the spine (55%), brain (26%), and knee (11%).^69^ Despite valuable contributions, further scientific work is required for empowering the infrastructures.

## Conclusion

Iran is an ancient country with outstanding medical knowledge in the medieval period, including neuroanatomy and neurologic diseases. Nonetheless, in the modern period, the previous knowledge was put aside and western science was considered as the reference of medical knowledge. Most of the papers of Iranian researchers related to neuroscience have been published in 2006 to 2008, when the neurology and neuropharmacology subfields had the highest trend of publications.^   68 ^

Currently, there are approximately 950 practicing neurologists in Iran, and several residents are trained annually. Besides, there are increasing numbers of physicians with neurological subspecialties in Iran that mostly have undertaken fellowships in the United States (US) and European countries. 

Given said that, we need to establish more fellowship programs in the major universities of Iran in the near future to provide better management of common neurological disorders within the country. All in all, as neurology has been separated as a distinct medical field in Iran since about 30 years ago, there is a need to pay greater attention to this critical field of medicine. Increasing the number of neurology training programs, research facilities, and powerful infrastructures can bring a brighter future for neurology in Iran.

## Notes:

**How to cite this article:** Kant-Mishra S, Khanli HM, Akhlaghipour G, Ahmadi-Jazi G, Khosa S. Historical perspective of neurology in Iran. Iran J Neurol 2019; 7(1): 25-32.

## References

[1] Fisher WB (2013). The middle east (Routledge revivals): A physical, social and regional geography.

[2] Barthold VV (2014). An historical geography of Iran.

[3] Kashani-Sabet F (2000). Frontier Fictions: Shaping the Iranian Nation, 1804-1946.

[4] Curtis VS, Stewart S (2005). Birth of the Persian Empire: The Idea of Iran.

[5] Efthymiou-Egleton I "Wellness": A New Word for Ancient Ideas [Online].

[6] Amr SS, Tbakhi A (2007). Abu Bakr Muhammad Ibn Zakariya Al Razi (Rhazes): philosopher, physician and alchemist. Ann Saudi Med.

[7] Al-Ibri I ( 1992). Brief history Ad-Dual Li Ibn Al-Ibri.

[8] Amann R, Peskar BA (2002). Anti-inflammatory effects of aspirin and sodium salicylate. Eur J Pharmacol.

[9] Zargaran A, Mehdizadeh A, Zarshenas MM, Mohagheghzadeh A (2012). Avicenna (980-1037 AD). J Neurol.

[10] Behrouz R, Ourmazdi M, Reza'i P (1993). Iran the cradle of science.

[11] Calignano A, La Rana G, Giuffrida A, Piomelli D (1998). Control of pain initiation by endogenous cannabinoids. Nature.

[12] Elgood C (1932). A Medical history of Persia and the eastern caliphate: From the earliest times until the year A.D.

[13] Gorji A, Khaleghi Ghadiri M (2001). History of epilepsy in Medieval Iranian medicine. Neurosci Biobehav Rev.

[14] Gorji A, Kohling R, Straub H, Hohling JM, Madeja M (2001). Lowering the extracellular potassium concentration elicits epileptic activity in neocortical tissue of epileptic patients. Eur J Neurosci.

[15] Gorji A (2003). Pharmacological treatment of headache using traditional Persian medicine. Trends Pharmacol Sci.

[16] Broumand B (2005). Transplantation activities in Iran. Exp Clin Transplant.

[17] Lee Gordon B (1949). Medicine throughout antiquity.

[18] Browne EG (2008). Islamic medicine: Fitzpatrick lectures delivered at the Royal College of Physicians in 1919-1920.

[19] Bodjnordi KM (1996). The Great Islamic Encyclopedia.

[20] Al-Akhawyni Bokhari AB, Matini J (1965). Hidayat al-Muta`allemin Fi al-Tibb.

[21] Afshar A (2017). The Westminster Medical College and Hospital in Urmia, Iran, 1879-1915. Arch Iran Med.

[22] Bayan L, Modarres Mousavi SM, Gorji A (2013). History of neurological disorders in Persian medicine. Res Hist Med.

[23] Ebrahimnejad H (2002). Religion and medicine in Iran: from relationship to dissociation. Hist Sci.

[24] Nayernouri T (2015). A brief history of ancient Iranian medicine. Arch Iran Med.

[25] Filliozat J (1975). The classical doctrine of Indian medicine: Its origenes and greek parallels.

[26] Goodrich JT (2004). History of spine surgery in the ancient and medieval worlds. Neurosurg Focus.

[27] Nayernouri T (2010). Asclepius, Caduceus, and Simurgh as medical symbols; part II. Simurgh. Arch Iran Med.

[28] Shoja MM, Tubbs RS (2007). The history of anatomy in Persia. J Anat.

[29] Cultural Heritage News Agency (CHN) Burnt City woman’s face reconstructed [Online].

[30] Moghadasi AN (2014). Artificial eye in burnt city and theoretical understanding of how vision works. Iran J Public Health.

[31] Gharravi M, Orazizadeh M, Hashemitabar M, Ansari-Asl K, Banoni S, Alifard A (2012). Status of tissue engineering and regenerative medicine in Iran and related advanced tools: Bioreactors and scaffolds. J Biomed Sci Eng.

[32] Seyed Sajjadi SM (2008). First evidences on prehistoric surgery in Iran. Tourism Journal.

[33] (cited 2017). University of Gundi-Shapur first in the world [Online].

[34] Azizi MH (2008). Gondishapur School of Medicine: The most important medical center in antiquity. Arch Iran Med.

[35] Pourahmad J (2010). History of medical sciences in Iran. Iran J Pharm Res.

[36] Modanlou HD (2008). A tribute to Zakariya Razi. Arch Iran Med.

[37] Rhazes Web About Rhazes [Online].

[38] The Basic of Philosophy [Online].

[39] Mirzania M, Ghavamzadeh A, Asvadi Kermani I, Ashrafi F, Allahyari A, Rostami N (2015). Medical oncology, history and its future in Iran. Arch Iran Med.

[40] Azizi MH, Bahadori M, Dabiri S, Shamsi Meymandi S, Azizi F (2016). A History of Leishmaniasis in Iran from 19th Century Onward. Arch Iran Med.

[41] Sajadi MM, Sajadi MR, Tabatabaie SM (2011). The history of facial palsy and spasm: Hippocrates to Razi. Neurology.

[42] Keskinbora K, Keskinbora K (2016). A systematic review of Ibn Sina's (Avicenna) studies: Reflections on anatomy. Eur J Anat.

[43] Ekhtiar M (2001). Nasir al-Din Shah and the Dar al-Funun: The evolution of an institution-163. Iranian Studies.

[44] Wikipedia, the free encyclopedia Tehran University of Medical Sciences [Online].

[45] Scurlock JA, Andersen B (2005). Diagnoses in Assyrian and Babylonian medicine: Ancient sources, translations, and modern medical analyses.

[46] Zargaran A, Zarshenas MM, Karimi A, Yarmohammadi H, Borhani-Haghighi A (2013). Management of stroke as described by Ibn Sina (Avicenna) in the Canon of Medicine. Int J Cardiol.

[47] Zargaran A, Borhani-Haghighi A, Faridi P, Daneshamouz S, Mohagheghzadeh A (2016). A review on the management of migraine in the Avicenna's Canon of Medicine. Neurol Sci.

[48] Hamedi A, Zarshenas MM, Sohrabpour M, Zargaran A (2013). Herbal medicinal oils in traditional Persian medicine. Pharm Biol.

[49] Gorji A, Khaleghi Ghadiri M (2002). History of headache in medieval Persian medicine. Lancet Neurol.

[50] Amr SS, Tbakhi A (2007). Ibn Sina (Avicenna): The prince of physicians. Ann Saudi Med.

[51] Tibi S (2006). Al-Razi and Islamic medicine in the 9th century. J R Soc Med.

[52] Aciduman A, Belen D (2007). Hydrocephalus and its management in Avicenna's Canon of Medicine. J Neurosurg.

[53] Aciduman A, Belen D, Simsek S (2006). Management of spinal disorders and trauma in Avicenna's Canon of medicine. Neurosurgery.

[54] Ghaffari F, Naseri M, Movahhed M, Zargaran A (2015). Spinal Traumas and their Treatments According to Avicenna's Canon of Medicine. World Neurosurg.

[55] Bonakdarian M (2010). Iranian Studies in the United Kingdom in the Twentieth Century. Iranian Studies.

[56] Azizi MH, Azizi F (2010). Government-sponsored Iranian medical students abroad (1811-1935). Iranian Studies.

[57] Azizi MH (2012). Physicians in the first academy of Iran (1935-1953). Middle East J Dig Dis.

[58] Wikipedia the free encyclopedia Chehrazi E [Online].

[59] Tehran University of Medical Sciences Department of Neurology [Online].

[60] Tehran University of Medical Sciences Primates/Dr. Ebrahim Chahrazi, Professor of Brain &amp; Pain (Nerves) [Online].

[61] Saberi-Firoozi M, Mir-Madjlessi SH (2009). Development of gastroenterology and hepatology in Iran: part I-training programs and the Iranian association of gastroenterology and hepatology. Arch Iran Med.

[62] Iranianpath Biography of Dr. Jalal Barimani, the father of modern Iranian neurology and the founder of the Iranian Neuroscience and Clinical Neurophysiology Society [Online].

[63] Iranian Neurological Association Iranian Neurological Association, History and Introduction [Online].

[64] Iranian Journal of Neurology [Online].

[65] Wikipedia, the free encyclopedia Majid Samii [Online].

[66] Rikhtegar R, Zarrintan S (2014). Neurological letter from Iran. Pract Neurol.

[67] Lankarani KB, Alavian SM, Peymani P (2013). Health in the Islamic Republic of Iran, challenges and progresses. Med J Islam Repub Iran.

[68] Ashrafi F, Mohammadhassanzadeh H, Shokraneh F, Valinejadi A, Johari K, Saemi N (2012). Iranians' contribution to world literature on neuroscience. Health Info Libr J.

[69] Hossein-Zadeh A, Soltanian-Zadeh H (2010). Neuroimaging in Iran: A Review. Basic Clin Neurosci.

